# Real-World Efficacy of Upadacitinib in Patients With Ulcerative Colitis

**DOI:** 10.7759/cureus.87903

**Published:** 2025-07-14

**Authors:** Abdul Ghaffar, Fraz Ahmad, Joseph Collum, Linta Naveed

**Affiliations:** 1 Gastroenterology and Hepatology, East Lancashire Hospitals NHS Trust, Blackburn, GBR; 2 Internal Medicine, East Lancashire Hospitals NHS Trust, Blackburn, GBR; 3 Geriatrics, East Lancashire Hospitals NHS Trust, Blackburn, GBR

**Keywords:** crohn's disease, fecal calprotectin, inflammatory bowel disease, jak inhibitor, mayo score, ulcerative colitis, upadacitinib

## Abstract

Upadacitinib (UPA) is a selective JAK-1 inhibitor used in the treatment of ulcerative colitis (UC) and Crohn’s disease. We report a real-world retrospective study evaluating the efficacy and safety of UPA in 17 patients who scaled from mild to severe UC, and demonstrated significant improvement in Inflammatory biomarkers, with the majority (16/17 patients) achieving reduced fecal calprotectin levels. Three patients had subsequent endoscopic evaluation after being initiated on UPA; two showed improvement in the Mayo score, and the remaining patient remained static. The side effect profile was consistent with known JAK inhibitor effects, with elevated cholesterol as the most common adverse event, and one case of neutropenia was observed. These findings suggest that UPA is an effective therapeutic option for refractory UC patients, and further studies would help in confirming its long-term efficacy in these patients.

## Introduction

The medical therapies for ulcerative colitis (UC) and Crohn’s disease (CD) have expanded in recent years. This expansion has in part been driven by the emergence of small-molecule therapies, which offer novel, effective options as well as oral delivery with quick onset of action and no risk of immunogenicity. These advancements are leading to the development of targeted treatment strategies for Inflammatory bowel disease (IBD), hence establishing that we are moving into an era of disease-modifying treatments [1,2].

Janus kinase (JAK) inhibitors are a class of targeted small molecules that prevent the activation of signal transduction. Depending on which JAK is impacted, they help control inflammation in the gut, joints, and skin. Upadacitinib (UPA) is a second-generation JAK inhibitor that selectively targets JAK-1 [3]. It is believed that JAK-1 selectivity leads to a better balance of risks and benefits, and initially it was successfully used for rheumatoid arthritis, which opened the door for its use in IBD [4-6].

UPA was approved by the U.S. Food and Drug Administration (FDA) for use in moderately to severely active UC in March 2022 and was also advocated by the National Institute for Health and Clinical Excellence (NICE) in January 2023. In terms of dosing, an induction dose of 45 mg daily for eight weeks is suggested, and either 15 mg or 30 mg daily for maintenance [7]. The primary aim of this study was to assess the real-world effectiveness of UPA in patients with UC, measured by changes in inflammatory biomarkers and improvement in clinical symptoms, indicative of reduced disease activity. The secondary aim was to evaluate the safety profile of UPA in these patients by documenting the incidence and nature of any adverse events observed during treatment.

## Materials and methods

This was a retrospective, observational, single-center study conducted at Royal Blackburn Hospital, East Lancashire Hospitals NHS Trust, Blackburn, United Kingdom. It aimed to evaluate the real-world effectiveness and safety of UPA in 17 patients with UC who initiated treatment between February 2023 and July 2024. This study was conducted as part of a clinical service evaluation, utilizing data collected during routine clinical care, and is covered by the guidelines from the NHS Health Research Authority [8].

Inclusion and exclusion criteria

Patients included in the study were at least 18 years of age, had a confirmed diagnosis of UC based on clinical, endoscopic, and histological evaluation, and had a minimum of eight weeks of follow-up data available. Patients were excluded if they had a diagnosis of CD, indeterminate colitis, or if their medical records were incomplete or lacked the minimum follow-up period required after initiating UPA.

Data collection

Clinical data were collected from electronic medical records as part of routine clinical practice. This included patient demographics such as age, sex, and geographic region, the extent of disease involvement, prior use of biologics or immunomodulators, presenting clinical symptoms, and inflammatory biomarker measurements, primarily fecal calprotectin (FCP) levels recorded at baseline and after ≥8 weeks of treatment. Where available, endoscopic findings were also reviewed, using the Mayo Score or Disease Activity Index (DAI) to assess disease severity. Adverse events and safety profiles were documented, including any abnormal laboratory findings or reported clinical side effects during the treatment period.

Data analysis

Descriptive statistics were used to summarize the patient demographics, clinical characteristics, and outcomes. Categorical variables were presented as frequencies and percentages. The percentage reduction in FCP was calculated to measure the biochemical response.

## Results

**Figure 1:**
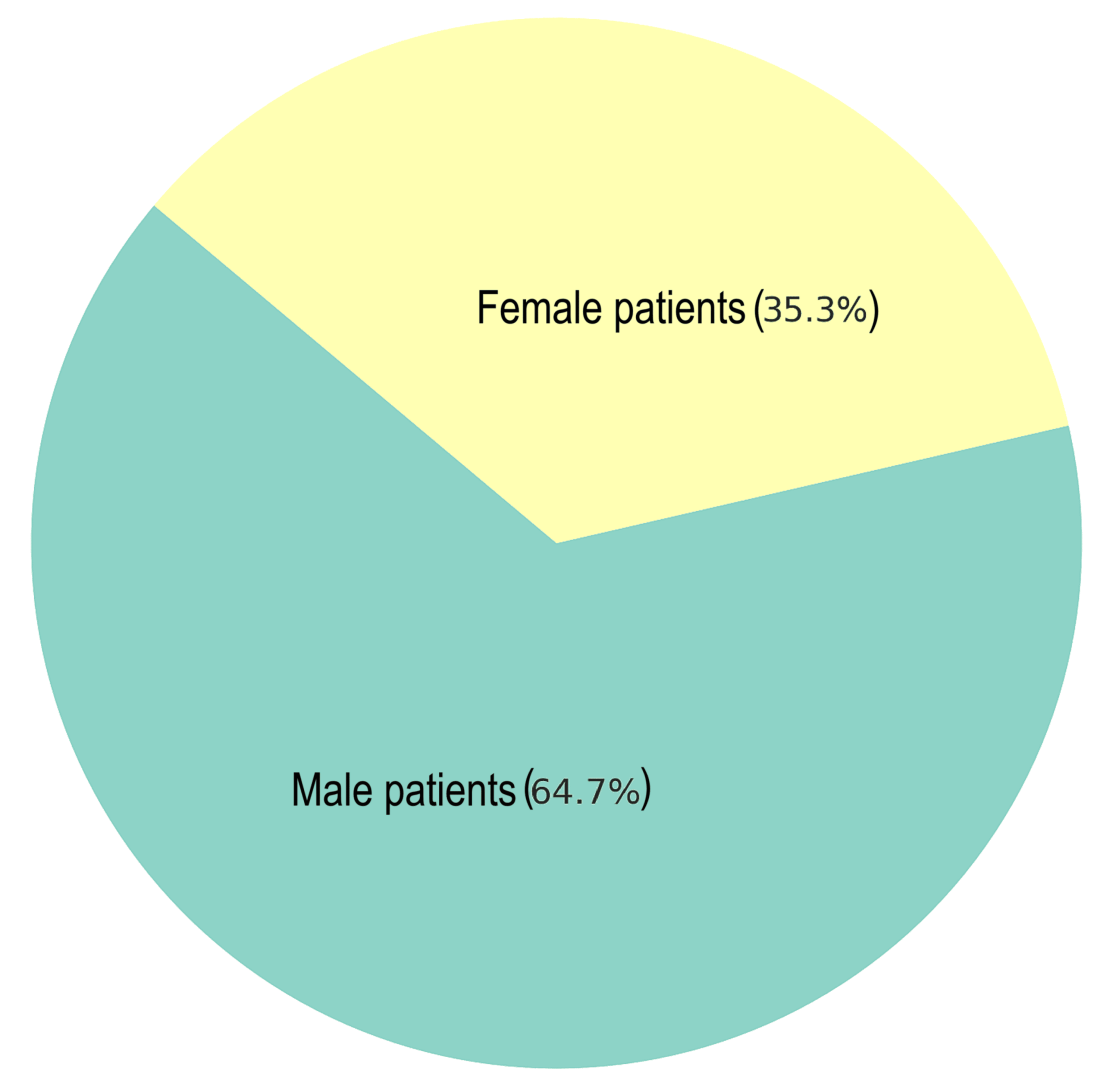
Gender distribution of patients

**Figure 2:**
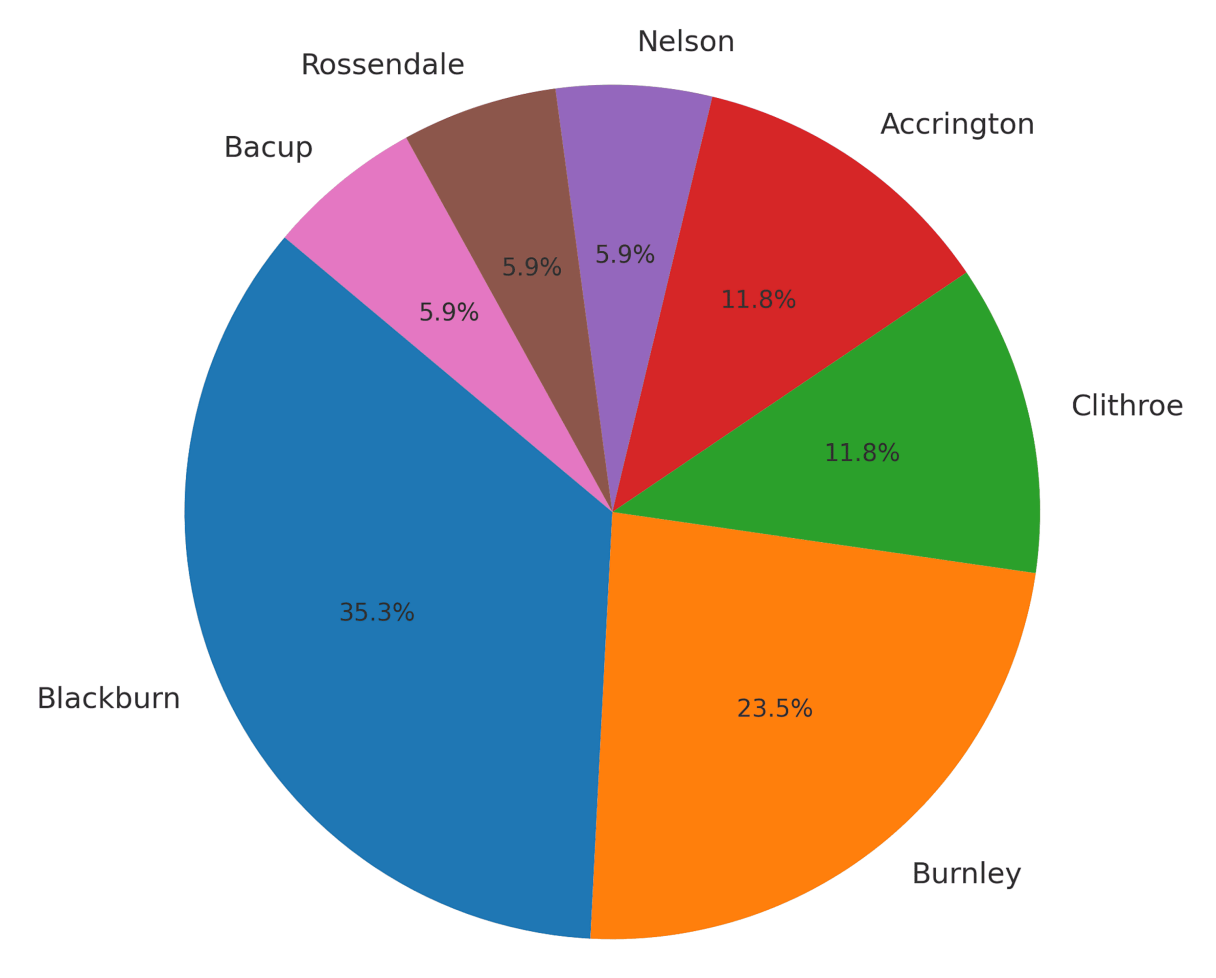
Distribution of patients as per demographic mapping

A total of 17 patients were included in the study. A gender distribution chart (Figure 1) reflected that patients were predominantly male, with only six (35.3%) female patients in the cohort. Demographic mapping (Figure 2) reflected that the majority of patients were from Blackburn and Burnley.

**Figure 3:**
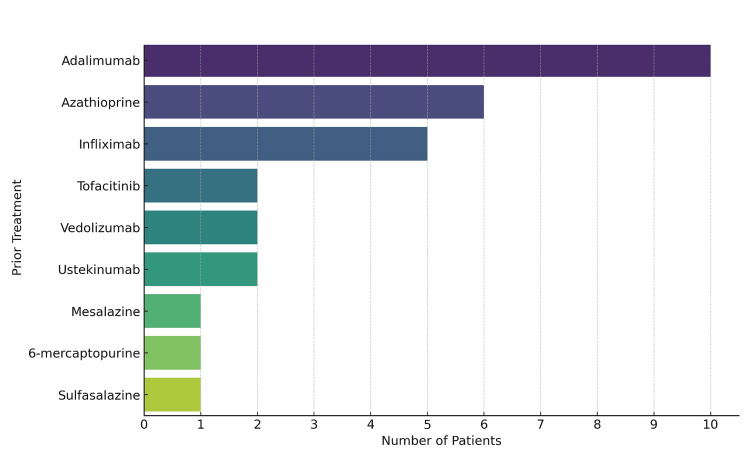
Frequency of biologic and immunomodulator treatments prior to being started on upadacitinib

An analysis of prior treatments (Figure 3) showed that most patients had previously received one or more therapies, including biologics such as infliximab, adalimumab, vedolizumab, and ustekinumab; a JAK inhibitor, tofacitinib; and conventional treatments such as azathioprine, 6-mercaptopurine, mesalazine, and sulfasalazine. Notably, eight patients (47.1%) had been exposed to two or more of these agents.

**Figure 4:**
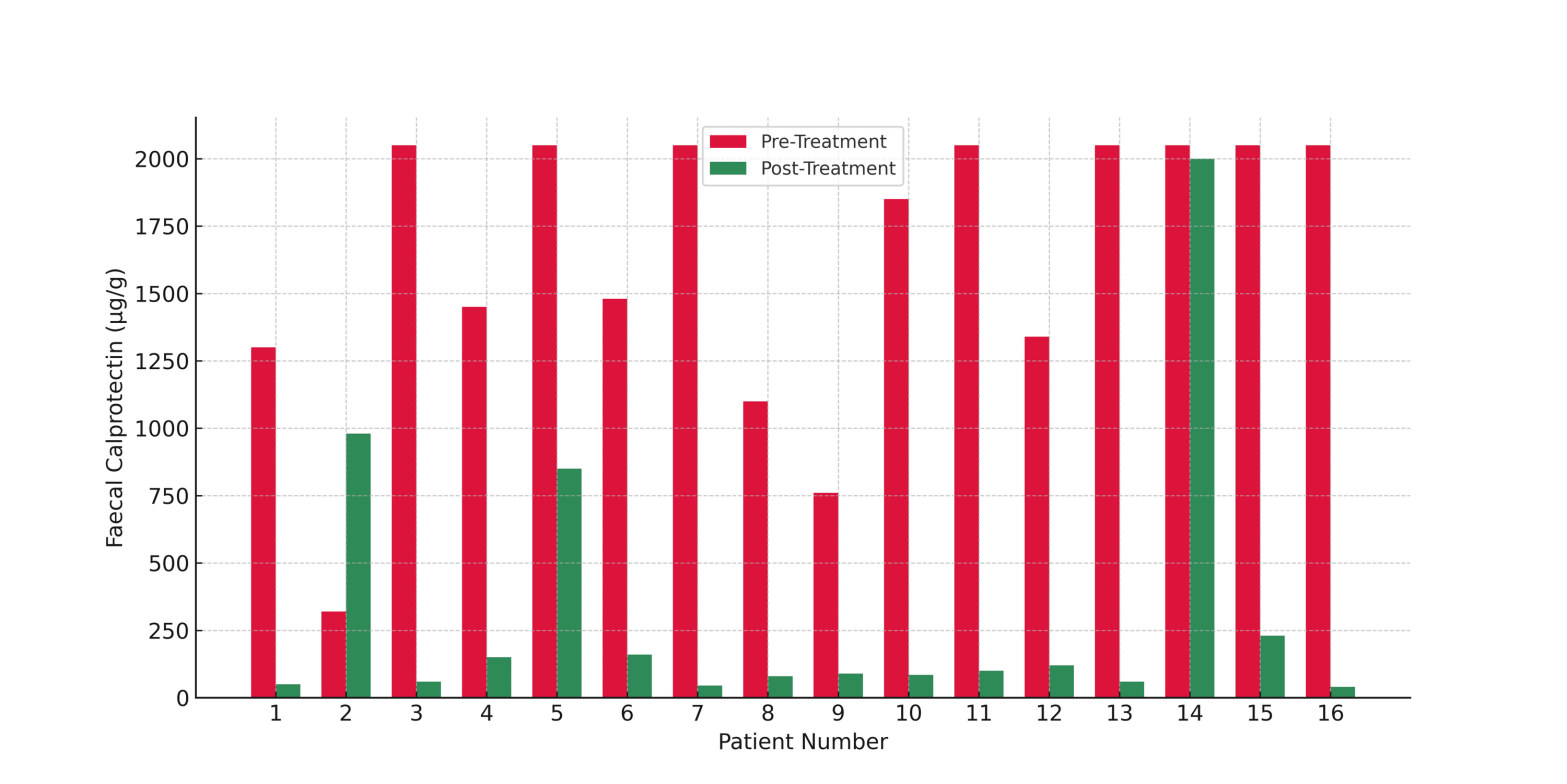
Fecal calprotectin levels before and after treatment with upadacitinib

**Figure 5:**
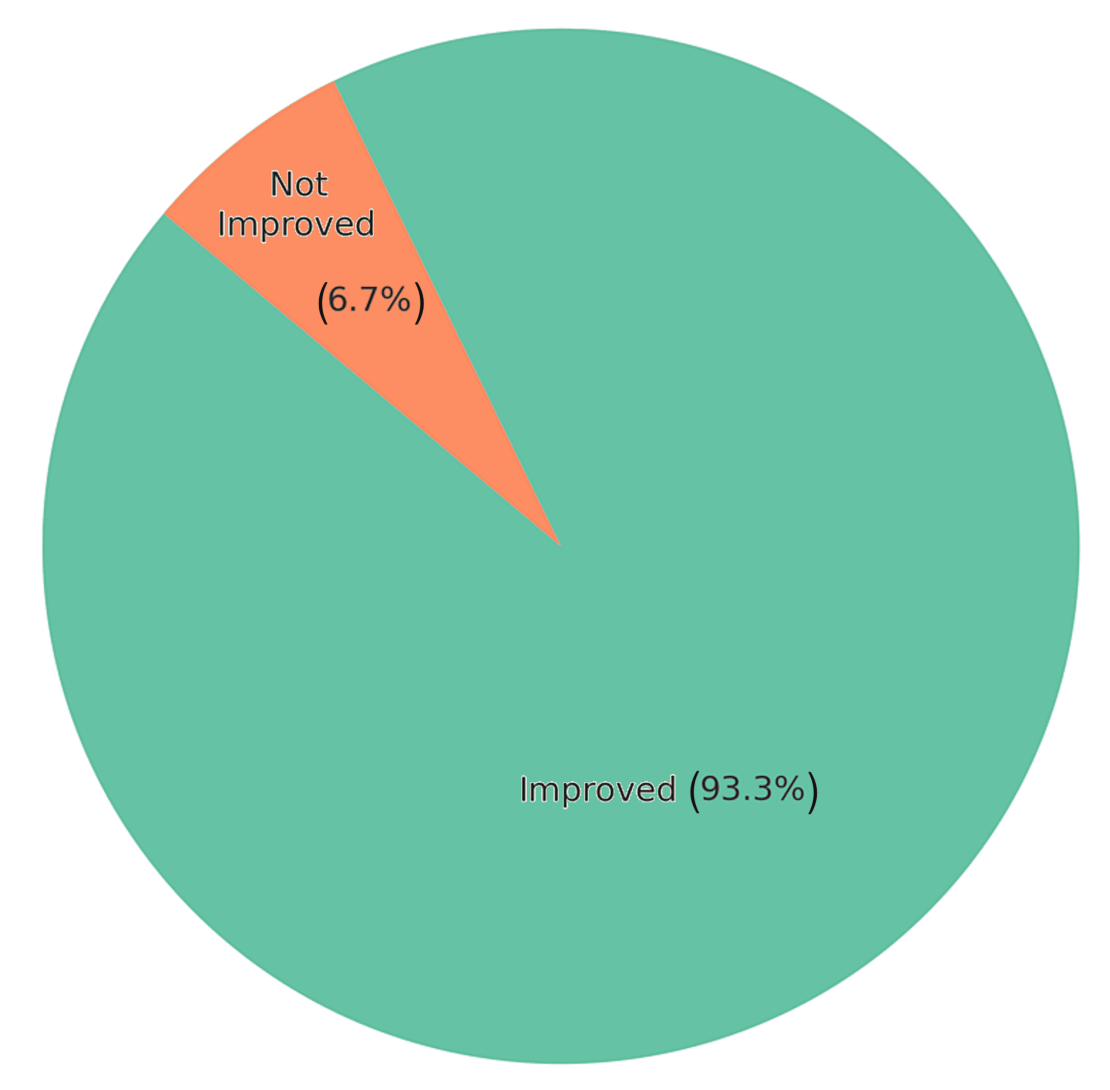
Overall FCL percentage of improvement among patients FCL: fecal calprotectin level

At follow-up after eight weeks or more, 14 (87.5%) patients demonstrated a marked reduction in FCP levels, with 13 (81.25%) patients showing a high level of biochemical response (≥75% reduction). Two patients exhibited no reduction in FCP levels, with corresponding worsening of clinical symptoms, suggesting disease progression despite UPA therapy (Figure 4). One additional patient, who had a recorded baseline FCP level of <15 µg/g and did not have a follow-up FCP measurement, was not included in the quantitative FCP analysis but was documented to have shown clear symptomatic improvement following treatment initiation with UPA. A significant majority of patients experienced clinical and symptomatic benefit after being treated with UPA, as reflected in Figure 5.

**Figure 6:**
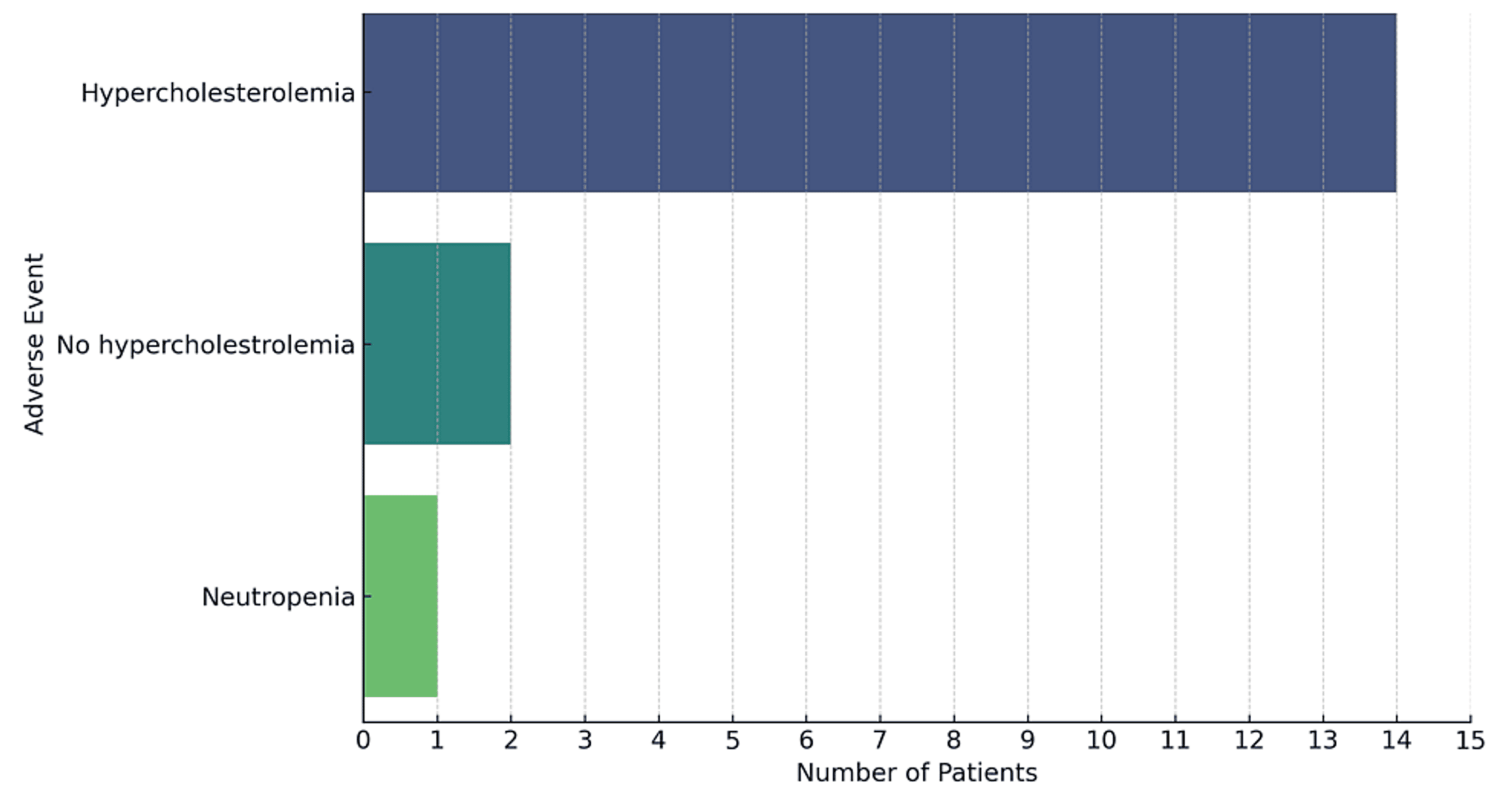
Frequency of adverse events among patients

All these patients with UC had received the FDA-approved induction dose for UPA, i.e., 45 mg once daily for eight weeks. Hypercholesterolemia was the most commonly recorded adverse event, observed in 14 (82.4%) patients. One patient also developed neutropenia. Three patients recorded no hypercholesterolemia or any other significant side effects related to UPA (Figure 6). 

## Discussion

This retrospective observational study evaluated the real-world use of UPA in a cohort of patients with mild to severe UC, the majority of whom had previously failed several advanced therapies. Overall, our analysis demonstrates that UPA has promising clinical and biochemical results.

A significant reduction in FCP was observed in the majority of patients, with 14 (87.5%) patients showing improvement, and the majority of patients achieving a substantial biochemical response (≥75% reduction in FCP). It's worth mentioning that all the patients showed clinical/symptomatic improvement when their FCP levels went down with therapy.

Since most of the patients showed improvement in their symptoms for UC after being started on UPA, endoscopic evaluation post treatment was not considered for all patients. Among the patients who underwent endoscopic evaluation, the Mayo Score/Disease Activity Index (DAI) for UC was used for evaluation of the disease. Among the three patients who underwent endoscopic reassessment, two demonstrated improvement in Mayo scores, thus further supporting the clinical efficacy of UPA in patients with UC. Although the number of endoscopic assessments was limited in our study, the percentage improvement noted aligns with the reports seen in emerging real-world literature on the use of UPA in UC [9].

It is notable that we didn't set a specific washout period for patients on previous biologic treatments before starting UPA. In the real world, it's essential to begin new biologic therapy sooner rather than later, as the patients are unwell and require earlier initiation of therapy. In a sense, the data also suggest that washout periods between biologic therapies may not be necessary.

This study also supports the use of UPA in patients who have not had any biologic therapies previously. Two of the patients included in this study fall into this category. One of these patients experienced significant improvement in FCP after being treated with UPA, whilst the other patient did not demonstrate any signs of disease remission. This was in accordance with NICE guidelines, which advocate the use of UPA as an option for treating moderately to severely active UC in adults when the condition has not responded well enough to conventional treatments [10].

All patients included in the study had received the FDA/NICE-approved induction dose of 45 mg daily for eight weeks, with no significant safety concerns reported. In terms of the side effects reported in this limited cohort of patients for UPA, the most frequently observed adverse event was hypercholesterolemia. This is consistent with well-reported side effects for JAK inhibitors [11]. One of the patients developed neutropenia; however, no serious infections, thromboembolic events, or other significant complications were reported during the follow-up period.

Our findings are consistent with both clinical trial data and emerging real-world studies evaluating the effectiveness of UPA in UC. The pivotal phase 3 maintenance study (U-ACHIEVE maintenance) for UPA reported sustained clinical remission in 42-52% of patients at 52 weeks, reinforcing UPA's long-term utility in UC [12]. A recent real-world study involving 44 patients with UC who were treated with UPA showed that 81.5% of the patients achieved clinical remission [13], which is comparable to results from our study. Another recent systematic review and meta-analysis of the comparative efficacy of biologics in UC, which analyzed around 36 existing studies and involved 14,270 patients with UC, also ranks UPA as the highest in inducing clinical remission with a percentage of about 99.6% [14], comparable to our data. 

Our experience with UPA has been significantly positive in achieving effective disease control in UC patients. It also demonstrates a reasonably effective safety profile, making it very useful for managing UC flares and reducing the dependence on corticosteroids.

Strengths and limitations

The core strength of this study is the promising high percentage of improvement in clinical symptoms, biochemical markers, and endoscopic evaluation seen in a significant majority of the patients commenced on UPA for UC.

The main limitation of this study was that it was a retrospective analysis in a single center, with a relatively small sample size. Additionally, endoscopic evaluation was not uniformly available in the cohort. We also acknowledge that, as with any retrospective real-world study, there is a possibility of residual confounding due to variations in prior therapies, disease severity, and comorbidities, which may influence individual outcomes despite our attempt to describe baseline characteristics in detail. Despite these limitations, the results from this study offer valuable real-world insight into the practical use of UPA in challenging patients with UC who have not responded to prior treatments.

## Conclusions

In this retrospective study, UPA was found to be effective and well tolerated in patients with UC who had previously not responded to other advanced biologic therapies. Most patients experienced significant reductions in inflammatory biomarkers (FCP) and improvement in clinical symptoms. There were no serious adverse events encountered in this cohort of patients.

The findings from this study further contribute to the ongoing real-world evidence supporting the use of UPA in UC. Further prospective studies with larger cohorts and long-term follow-up would be beneficial to understand the outcomes and long-term effects of treatment with UPA.
